# Bio-Inspired Neural Adaptive Control of a Small Unmanned Aerial Vehicle Based on Airflow Sensors

**DOI:** 10.3390/s18103233

**Published:** 2018-09-26

**Authors:** Zijun Ren, Wenxing Fu, Supeng Zhu, Binbin Yan, Jie Yan

**Affiliations:** 1 School of Astronautics, Northwestern Polytechnical University, Xi’an 710072, China; supengzh@nwpu.edu.cn (S.Z.); yanbinbin@nwpu.edu.cn (B.Y.); jyan@nwpu.edu.cn (J.Y.); 2 Unmanned System Research Institute, Northwestern Polytechnical University, Xi’an 710072, China; wenxingfu@nwpu.edu.cn

**Keywords:** UAV, bio-inspired flight control, neural network, pressure sensor

## Abstract

Inspired by the exceptional flight ability of birds and insects, a bio-inspired neural adaptive flight control structure of a small unmanned aerial vehicle was presented. Eight pressure sensors were elaborately installed in the leading-edge area of the forward wing. A back propagation neural network was trained to predict the aerodynamic moment based on pressure measurements. The network model was trained, validated, and tested. An adaptive controller was designed based on a radial basis function neural network. The new adaptive laws guaranteed the boundedness of the adaptive parameters. The closed-loop stability was analyzed via Lyapunov theory. The simulation results demonstrated the robustness of the bio-inspired flight control system when subjected to measurement noise, parametric uncertainties, and external disturbance.

## 1. Introduction

With the development of science and technology, unmanned aerial vehicles (UAVs) are becoming increasingly popular in business and daily life. Although small unmanned aerial vehicles (SUAVs) provide a new perspective of social life in areas such as agriculture [1], industry [2], public security [3], package delivery [4,5], as well as entertainment and media [6], as a multipurpose tool its potentiality has not been fully tapped—the flight safety of SUAVs being one of the main constraints. The complexity of the aerodynamics of the atmospheric boundary layer increases rapidly with decreasing altitude. The flow field changes frequently because of changeable weather conditions and complex interactions between ground objects. Turbulence intensities near the ground can reach >40% and >15% in suburban and urban environments, respectively [7,8,9]. Severe turbulence can degrade the flight safety of SUAVs particularly in complex urban environments, which leads to their limited application. Traditional attitude control systems of SUAVs based on the low-cost and rough IMUs (inertial measurement units) and actuators can barely maintain flight stability.

However, birds and insects found commonly in everyday life demonstrate excellent flight skills. SUAVs, birds, and insects largely fly at low Reynolds number, where nonlinearity and separation occur [10]. It seems that problems caused by low Reynolds numbers do not bring a great deal of distress to flying animals. Previous research has revealed that flying living beings have the ability to sense the flow information around them [11,12,13,14]. Feathers in the wings of birds have a sensing mechanism to measure airspeed and to detect stall and separation [12]. The mechanosensory feather system of a bird can greatly reduce reaction time, and is advantageous for rapid maneuvering. It is still unknown how the avian brain processes signals generated from thousands of mechanoreceptors [13]. 

Inspired by the mechanism, interesting studies have been published which try to use flow information to enhance flight safety. Sergio et al. modified two SUAV platforms, one equipped with 12 strain gauge sensors and the other with an array of pressure sensors. Experimental results verified that force sensing and flow sensing offered advantages beyond IMUs [15]. Shen et al. proposed an algorithm to estimate the aerodynamic forces based on airflow measurement and designed a robust sliding mode controller. The simulation results demonstrated an enhanced performance of the control framework [16]. Mohamed et al. took a closer look at the interrelation between atmospheric turbulence and wing surface pressure variations. Wind tunnel experiments revealed that a high correlation appeared in the wing leading-edge region, making this a better choice of where to place pressure sensors [17]. Pressure sensors were also used to augment roll motion stability [18]. 

In order to enhance the flight stability of flying vehicles under parametric uncertainties and external disturbances, scholars and researchers have proposed numerous model-based control strategies [19,20]. Adaptive control and robust control are popular tools to deal with uncertainties [21,22], among which the global approximation attribute of the neural network (NN) is attracting scholars’ interest [23,24,25,26]. Zeghlache et al. [24] designed a fault-tolerant NN controller based on a faulty octorotor aerial vehicle model, which showed perfect tracking performance despite a rotor failure. The radial basis function neural network (RBF NN) was exploited to estimate external disturbances [23] and uncertain terms [26]. In [25], the authors incorporated a higher-order NN in a discrete backstepping scheme.

In this paper, a bio-inspired flight control framework is studied, where pressure sensors are integrated into the framework. The rest of the paper is organized as follows: in Section 2, the configuration of the pressure sensors of the test-model SUAV is presented, a back propagation neural network (BP NN) model is trained, validated, and tested, and a modified control-oriented model is proposed. The neural adaptive controller design and stability analysis are presented in Section 3. Finally, the simulation results and conclusions are shown in Section 4 and Section 5, respectively. 

## 2. Airflow Sensor System and Modeling

### 2.1. Pressure Sensor Configuration

Flying animals such as birds and insects have abundant mechanoreceptors in the wings and head to perceive airflow information around their bodies [12,27]. The anatomical features of pigeons demonstrate that about 70% of all the Herbst corpuscles are situated in the leading-edge region of the alula, and 30% are around the caudal band, yet there are few in the middle [11]. The density distribution of the Herbst corpuscles indicates where to measure the airflow information [13]. In order to obtain the flow field information around the SUAV, pressure sensors were selected and embedded in the front of the main wing. Previous research was also inclined to detect the airflow in the leading-edge area [15,17,18]. 

**Figure 1:**
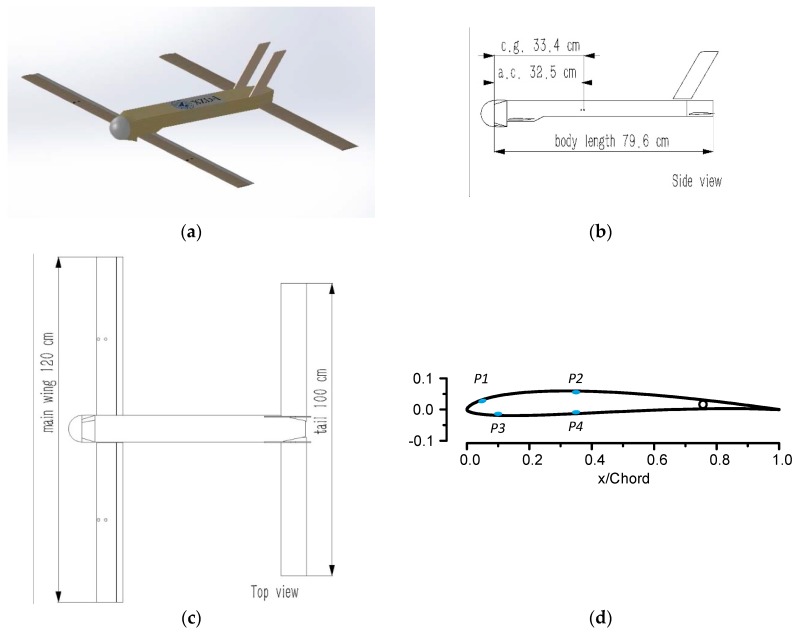
Small unmanned aerial vehicle (SUAV) model and pressure sensor configuration: (**a**) the XZD-I SUAV; (**b**) positions of the center of gravity (c.g.) and aerodynamic center (a.c.); (**c**) top view of the SUAV; (**d**) site of the sensors in the airfoil section.

A tandem-wing SUAV prototype named XZD-I was chosen as the model plane with which to conduct the experiments. Its wingspan was 1.2 m, its gross mass was 4 kg, and the designed trim velocity was 25 m/s (shown in Figure 1). Considering the sensor wiring and dimensional limitation, only eight sensors were installed in the front wing. Nos. 1–4 were in the middle of the left wing, Nos. 5–8 in the middle of right wing, symmetrically. Nos. 1–2 and 5–6 were placed on the upper wing surface, while Nos. 3–4 and 7–8 were on the lower wing surface (refer to Table 1 and Figure 1). 

### 2.2. Pitching Moment Prediction

Inspired by young birds learning to fly, a BP NN was introduced to build the complicated relationship between the pressure sensor measurements and the aerodynamic moment. 

#### 2.2.1. BP NN Modeling

A BP NN usually contains an input layer, an output layer, and one or more hidden layers. A BP NN of one hidden layer with enough neuron nodes can approximate any nonlinear function. The number of input and output layer nodes of the BP NN is related to the practical application. In this work, a BP NN with a three-layer structure was adopted. The input layer had eight nodes and the output layer had one node. The number of hidden nodes was calculated according to: $n_{hd}=\sqrt{n_{in}+n_{out}}+m$, where $n_{hd}$ is the number of hidden layer nodes, $n_{in}$ is the number of input layer nodes, $n_{out}$ is the number of output layer nodes, and m is a constant between 1 and 10. 

After several rounds of training experiments, it was better to increase the number of hidden layer nodes to ten. The BP NN was built and trained in MATLAB^®^ software. The tansig transfer function was assigned to the hidden layer, and the purelin function to the output layer. 

**Figure 2:**
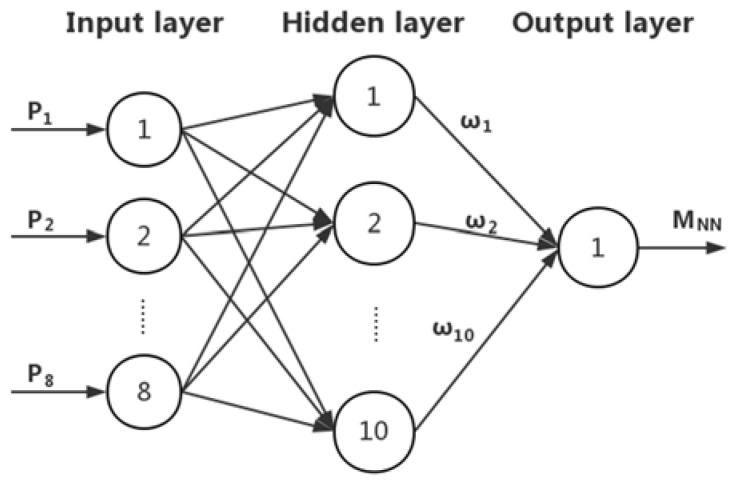
The back propagation neural network (BP NN) structure.

The network is shown in Figure 2. Eight pressure sensors’ outputs (P1–P8) were inputted to the eight input layer nodes. $M_{NN}$ was the pitching moment estimation output, and $\tilde{M}=M-M_{NN}$ was defined as the NN prediction error.

#### 2.2.2. Sample Preparation

In order to train the network, training and testing samples were constructed using the basic computational data of the SUAV model. The basic aerodynamic data were calculated in a widely used CFD code—CFL3D. The range of the attack angle *α* was chosen as −6° to 12°, which was an acceptable attitude envelope. Table 2 shows a representative aerodynamic data with a sampling rate of 2°. Because of the symmetry of the SUAV, the computational pressure outputs of sensors P5–P8 were nearly equal to P1–P4, respectively. Therefore, columns P5–P8 in the table were omitted for conciseness. Each line in Table 2 was a sample. *α* was the sample index. P1–P8 were the eight network inputs. M was the target. 

**Figure 3:**
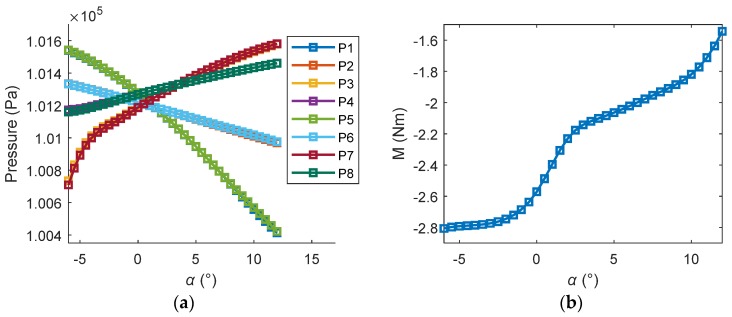
The computational data (sampling rate of 0.5°): (**a**) eight sensors’ measurements; (**b**) target data of pitching moments.

Figure 3 shows the computational sample data set. The computational sample set consisted of 37 groups of sampling data where the sample index *α* array was [−6 −5.5 −5 −4.5···11.5 12]. To improve the prediction accuracy of the network on noisy measurements, white noise was added to the computational data sample set. Ninety-nine noisy sample sets were randomly generated by adding normally distributed random numbers to the computational sample set. The random numbers were from a normal distribution with a mean of 0 and standard deviation 15, which simulated the measurement accuracy of ±15 Pa. 

The 100 sample sets (1 computational sample set and 99 noisy sample sets) served as network training inputs. The first 1–60 sample sets were the training sets, the following 61–80 sets were the validation sets, and the last 81–100 sets were the test sets. 

#### 2.2.3. Training and Testing the BP NN

Once the network and the sample sets were ready, the MATLAB^®^ Neural Network Toolbox was employed to train the network. The training function was the trainlm function. The maximal iteration times were 1000, the target error goal was $5\times 10^{-5}$, and the performance function was mean squared error (MSE). The remaining training parameters were the default values. 

**Figure 4:**
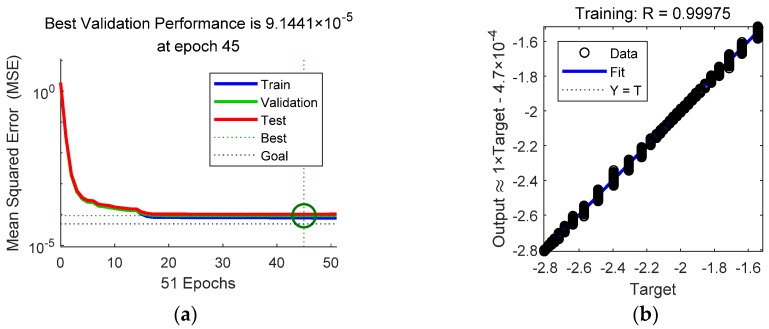

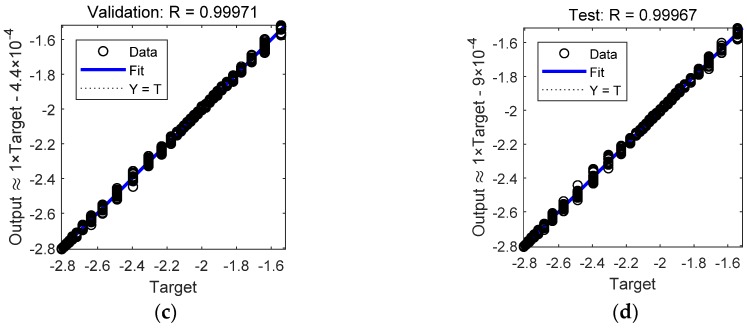
The BP NN training results: (**a**) the training errors of the training set, validation set, and test set; (**b**) regression of the training set; (**c**) regression of the validation set; (**d**) regression of the test set.

The training results are shown in Figure 4. The best training performance was $7.76\times 10^{-5}$. It seems that the training performance failed to achieve the target error goal, but it was still good. The regressions of the training set, validation set, and test set were 0.99975, 0.99971, and 0.99967, respectively. 

In order to test the prediction accuracy of the network, 36 samples of noisy data were chosen for the experiment. A new set of aerodynamic data between sampling intervals was recalculated in CFL3D to test the performance. Table 3 shows the index of the test samples. 

**Figure 5:**
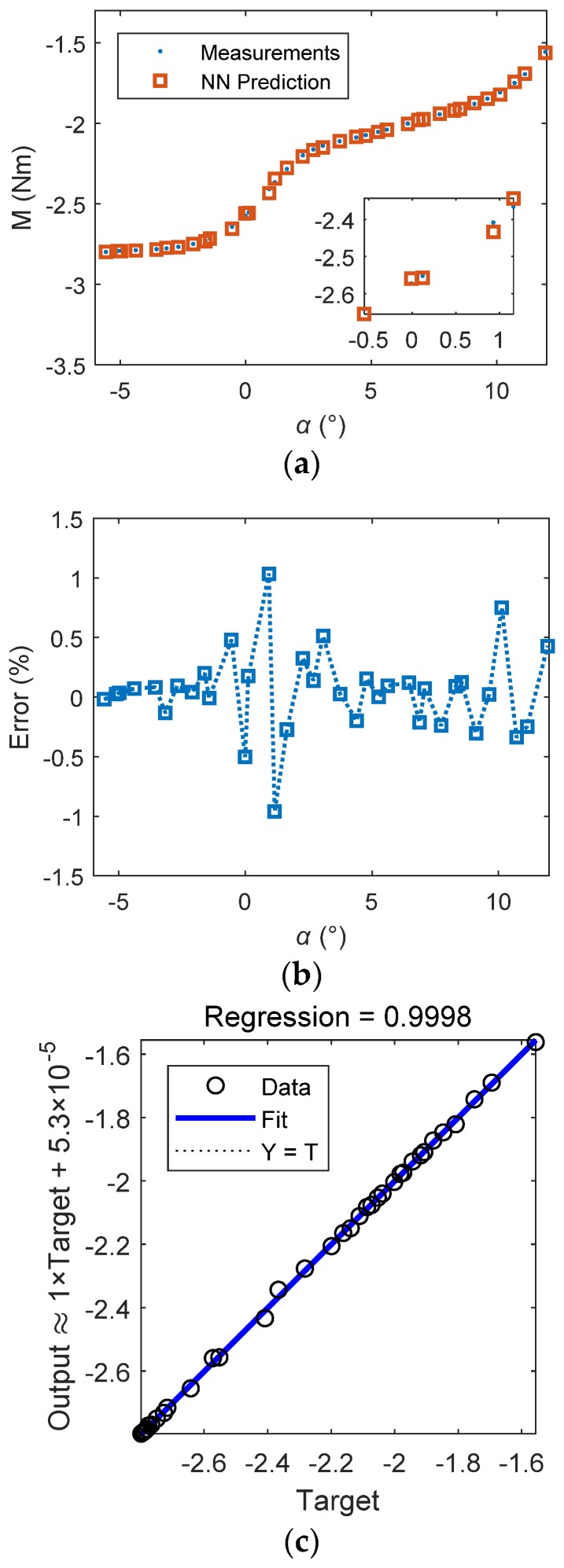
The BP NN test results: (**a**) comparison of measurements and NN prediction; (**b**) the prediction error; (**c**) regression of test samples.

The test results are shown in Figure 5. The prediction values and the measured values matched well. The prediction errors were between ±1.5%, which showed the high accuracy of the BP NN model for the pitching moment prediction.

### 2.3. Control-Oriented Modeling

The longitudinal static instability of the XZD-I SUAV places greater demands on the attitude controller design. In this paper, the pitching channel stabilization was taken as the example to illustrate the bio-inspired control architecture. The typical method of modeling the pitching moment is to linearize $C_{M}\left(\alpha ,\delta_{e}\right)$ as $C_{M}^{0}+C_{M}^{\alpha }\cdot \alpha +C_{M}^{\delta_{e}}\cdot \delta_{e}$, where $C_{M}^{*}$ are aerodynamic coefficients, α is the angle of attack, and $\delta_{e}$ is the elevator angular deflection. Nevertheless, the pitching moment of the SUAV is nonlinear about α and $\delta_{e}$, depicted in Figure 3. The typical model will cause a large modeling error, which results in a large control gain and a low stability margin.

The flow field around the leading-edge area is less affected by the deflection of the trailing edge flap for a wing of infinite span [28]. It is reasonable that pressure sensors P1–P8 in the leading-edge domain can be exploited to estimate the basic aerodynamic forces (zero flap angle). In order to take full advantage of the airflow information, we present a modified model of the pitching moment.

**Modified pitching moment model**

The pitching moment is given as follows:

(1) $$C_{M}\left(\alpha ,\delta_{e}\right)=C_{M}\left(\alpha \right)|{}_{\delta_{e}=0}+C_{M}^{\delta_{e}}\left(\alpha ,\delta_{e}\right)\cdot \delta_{e}+\Delta C_{M},$$

where $C_{M}\left(\alpha \right)|{}_{\delta_{e}=0}$ = $C_{M}\left(\alpha ,\delta_{e}=0\right)$ can be estimated through pressure information, and $\Delta C_{M}$ is the modeling error. The longitudinal kinetic equations can be written as:

(2) $$\begin{array}{l} \dot{\theta }=q\\ I_{yy}\cdot \dot{q}=M\left(\alpha \right)|{}_{\delta_{e}=0}+M^{\delta_{e}}\left(\alpha ,\delta_{e}\right)\cdot \delta_{e}+\Delta M+D_{T} \end{array}$$

where $I_{yy}$ is the moment of inertia, θ is pitch angle, q is pitch angular rate, $M=\bar{q}S_{ref}L_{ref}C_{M}$ is the pitching moment, and $D_{T}$ is the external disturbance torque.

During the trim flight condition, an assumption is made on flight path angle that γ≈0. Hence, the longitudinal angle equation θ=α+γ becomes θ≈α. The angle of attack can be replaced by the pitch angle θ for a short period during a trim flight.

Define $x_{1}=\theta$, $x_{2}=q$. The longitudinal kinetic Equation (2) can be rewritten as a more general strict feedback form:

(3) $$\begin{array}{l} {\dot{x}}_{1}=f_{1}\left(x_{1}\right)+g_{1}\left(x_{1}\right)\cdot x_{2}\\ {\dot{x}}_{2}=f_{2}\left(x_{1},x_{2}\right)+g_{2}\left(x_{1},x_{2}\right)\cdot u+d_{2} \end{array}$$

where $f_{1}=0$, $g_{1}=1$, $f_{2}=\left(M\left(\alpha \right)|{}_{\delta_{e}=0}+\Delta M\right)/I_{yy}$, $g_{2}=M^{\delta_{e}}\left(\alpha ,\delta_{e}\right)/I_{yy}$, $d_{2}=D_{T}/I_{yy}$. $M\left(\alpha \right)|{}_{\delta_{e}=0}={\hat{M}}_{N}+\tilde{M}$, where ${\hat{M}}_{N}$ is the BP NN output $M_{NN}$ and $\tilde{M}$ is the estimation error. Considering the uncertainties and external perturbation, Equation (3) becomes:

(4) $$\begin{array}{l} {\dot{x}}_{1}=f_{1}+g_{1}\cdot x_{2}\\ {\dot{x}}_{2}=f_{2N}+g_{2}\cdot u+d \end{array}$$

where $f_{2N}={\hat{M}}_{N}/I_{yy}$, $d=\left(\tilde{M}+\Delta M+D_{T}\right)/I_{yy}$.

**Assumption** **1.** *There exist constants*${\underline{g}}_{2}$*and*${\bar{g}}_{2}$*such that*$0<{\underline{g}}_{2}\leq g_{2}\leq {\bar{g}}_{2}$*. External disturbance torque*$D_{T}$*is assumed to be bounded and change slowly*.

The control goal is to synthesize a bounded control signal u to drive the actual pitch angle $x_{1}\left(t\right)$ to the desired angular trajectory $x_{1d}\left(t\right)$ as closely as possible, in spite of model uncertainties and external disturbance.

## 3. Neural Adaptive Controller

### 3.1. RBF NN

To achieve the control goal, neural network and adaptive approximation approaches were applied. It was proved that RBF NN has the ability of universal approximation [29,30]. A commonly used Gaussian function RBF NN is simply described as [20]:

(5) $$y_{NN}=\omega^{T}h\left(x\right),$$

where $\omega \in R^{l}$ is weight vector, l is the number of hidden layer nodes, and $h\left(x\right)={\left[h_{1}\left(x\right),h_{2}\left(x\right),\cdots h_{l}\left(x\right)\right]}^{T}\in R^{l}$ is the basis function vector. The Gaussian function $h_{i}\left(x\right)$ has the form:

(6) $$h_{i}\left(x\right)=\exp\left(-\frac{{\left\|x-\mu_{i}\right\|}^{2}}{\sigma_{i}^{2}}\right)\;,$$

where $\mu_{i}$ is the *i*th center vector of the receptive field, and $\sigma_{i}$ is the *i*th width of the Gaussian function.

### 3.2. Controller Design

The synthetic controller was designed via a backstepping structure.

Define the tracking error as:

(7) $$z_{1}=x_{1}-x_{1d}.$$

Following the backstepping design schemes, select Lyapunov function $V_{1}$ as:

(8) $$V_{1}=\frac{1}{2}z_{1}^{2}.$$

The derivative of $V_{1}$ along the trajectories of system (4) is:

(9) $${\dot{V}}_{1}=z_{1}\left(f_{1}+g_{1}x_{2}-x_{1d}\right).$$

Taking $x_{2}$ as the virtual control to stabilize the subsystem ${\dot{x}}_{1}=f_{1}+g_{1}\cdot x_{2}$ (where $f_{1}=0$, $g_{1}=1$), an alternative desired virtual control value $x_{2d}$ is selected as:

(10) $$x_{2d}=-c_{1}z_{1}+{\dot{x}}_{1d},$$

where $c_{1}>0$ is the control gain of the first subsystem. Defining $z_{2}=x_{2}-x_{2d}$ and substituting $z_{2}$ into Equation (9), yields:

(11) $${\dot{V}}_{1}=-c_{1}z_{1}^{2}+z_{1}z_{2}.$$

If $x_{2}$ follows the track of $x_{2d}$ closely, indicating that $z_{2}\approx 0$, $V_{1}$ will exponentially decay. Define a Lyapunov function $V_{2}$ as:

(12) $$V_{2}=\frac{1}{2}z_{2}^{2}.$$

In view of Equations (4), (11), and (12), the derivative of $V_{1}+V_{2}$ is:

(13) $${\dot{V}}_{1}+{\dot{V}}_{2}=-c_{1}z_{1}^{2}+z_{2}\left(z_{1}+f_{2N}+g_{2}u+d+c_{1}{\dot{z}}_{1}-{\ddot{x}}_{1d}\right).$$

Since $g_{2}$ and d are unknown, the task is to design an adaptive controller u and update laws ${\dot{\hat{g}}}_{2}$ and $\dot{\hat{d}}$, satisfying $0<{\underline{g}}_{2}<{\hat{g}}_{2}<{\bar{g}}_{2}$. We design the adaptive control law:

(14) $$u=\frac{1}{{\hat{g}}_{2}}\left(-f_{2N}+{\ddot{x}}_{1d}-z_{1}-c_{1}{\dot{z}}_{1}-c_{2}z_{2}-\hat{d}\right),$$

where ${\hat{g}}_{2}$ and $\hat{d}$ are the estimations of $g_{2}$ and d, respectively. $c_{2}>0$ is the control gain of the second subsystem. The update law ${\dot{\hat{g}}}_{2}$ has the form:

(15) $${\dot{\hat{g}}}_{2}=proj.\left(\eta z_{2}u-\eta \sigma \left|z_{2}\right|\left({\hat{g}}_{2}-g_{2N}\right)\right),$$

where η>0, σ is a small positive value, and $g_{2N}$ is the nominal value of $g_{2}$. proj.(χ) is the projection modification defined as follows:

(16) $$proj.\left(\chi \right)=\{\begin{array}{ll} 0, & \mathrm{Case}-I:\;\mathrm{if}\;{\hat{g}}_{2}\geq {\bar{g}}_{2}\;\mathrm{and}\;\chi \geq 0\\ 0, & \mathrm{Case}-\mathrm{II}:\;\mathrm{if}\;{\hat{g}}_{2}\leq {\bar{g}}_{2}\;\mathrm{and}\;\chi \leq 0\\ \chi , & \mathrm{Case}-\mathrm{III}:\;\mathrm{otherwise} \end{array}\;$$

Note that the projection process ensures ${\underline{g}}_{2}\leq {\hat{g}}_{2}\leq {\bar{g}}_{2}$, provided that ${\hat{g}}_{2}\left(t_{0}\right)\in \left[{\underline{g}}_{2},{\bar{g}}_{2}\right]$, which guarantees non-singularity of the control law u over the considered flight envelope. The composite uncertainty d is approximated by RBF NN:

(17) $$\begin{array}{c} d=d^{*}+\varepsilon_{d}\\ d^{*}=\omega^{*T}\cdot h\\ \hat{d}={\hat{\omega }}^{T}\cdot h \end{array}$$

where $d^{*}$ is the ideal approximation of RBF NN with a finite number of hidden layer nodes, $\omega^{*}$ is the ideal output layer weights vector (${\left\|\omega^{*}\right\|}_{2}^{2}\leq \omega_{M}$, $\omega_{M}$ is a positive constant [20]), h is the output vector of the hidden layer, $\hat{d}$ is the updated estimation, $\hat{\omega }$ is the updated output layer weights vector, and $\varepsilon_{d}$ is the approximation error ($\left|\varepsilon_{d}\right|\leq \varepsilon_{M}$, $\varepsilon_{M}>0$ denotes the supremum of the approximation error [20,31]). The adaptive law of $\hat{\omega }$ is designed as:

(18) $$\dot{\hat{\omega }}=\gamma z_{2}h-\gamma \nu \left|z_{2}\right|\hat{\omega },$$

where γ and ν are designed positive parameters.

**Figure 6:**
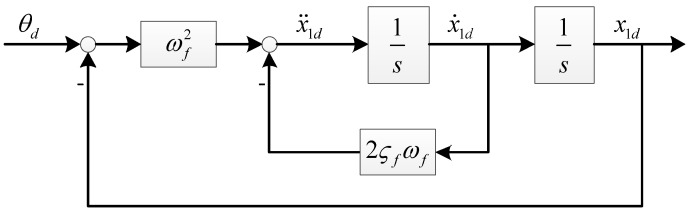
Command pre-filter.

**Remark** **1.** *The desired pitch signal*$\theta_{d}$*is filtered by a second-order pre-filter (refer to Figure 6).*$x_{1d}$*,*${\dot{x}}_{1d}$*, and*${\ddot{x}}_{1d}$*are generated by the pre-filter. The parameters of the pre-filter were chosen as*$\omega_{f}=4$*and*$\varsigma_{f}=0.9$.

### 3.3. Stability Analysis

Define Lyapunov function candidates $V_{g}$ and $V_{d}$:

(19) $$V_{g}=\frac{1}{2\eta }{\tilde{g}}_{2}^{2}<\mathrm{obj}/>,\;V_{d}=\frac{1}{2\gamma }{\tilde{\omega }}^{T}\tilde{\omega },$$

where ${\tilde{g}}_{2}=g_{2}-{\hat{g}}_{2}$ and $\tilde{\omega }=\omega^{*}-\hat{\omega }$.

Define $V=V_{1}+V_{2}+V_{g}+V_{d}$, thus the derivative of V along the trajectory of the system (4) is:

(20) $$\begin{array}{cc} \dot{V} & ={\dot{V}}_{1}+{\dot{V}}_{2}+{\dot{V}}_{g}+{\dot{V}}_{d}\\ & =-c_{1}z_{1}^{2}-c_{2}z_{2}^{2}+z_{2}{\tilde{\omega }}^{T}h+z_{2}\varepsilon_{d}+z_{2}u{\tilde{g}}_{2}+\frac{1}{\eta }{\tilde{g}}_{2}\left(-{\dot{\hat{g}}}_{2}\right)+\frac{1}{\gamma }{\tilde{\omega }}^{T}\left(-\dot{\hat{\omega }}\right)\\ & =-c_{1}z_{1}^{2}-c_{2}z_{2}^{2}+\frac{1}{\eta }{\tilde{g}}_{2}\left(\eta z_{2}u-{\dot{\hat{g}}}_{2}\right)+\frac{1}{\gamma }{\tilde{\omega }}^{T}\left(\gamma z_{2}h-\dot{\hat{\omega }}\right)+z_{2}\varepsilon_{d} \end{array}$$

Substituting (15), (16), and (18) into (20), $\dot{V}$ can be rewritten as:

(21) $$\dot{V}=\underset{1}{\underset{︸}{\begin{array}{c} -c_{1}z_{1}^{2} \end{array}}}\underset{2}{\underset{︸}{\begin{array}{c} -(c_{2}z_{2}^{2}-z_{2}\varepsilon_{d}) \end{array}}}+\underset{3}{\underset{︸}{\begin{array}{c} \nu \left|z_{2}\right|{\tilde{\omega }}^{T}\hat{\omega } \end{array}}}+\underset{4}{\underset{︸}{\{\begin{array}{ll} {\tilde{g}}_{2}z_{2}u, & \mathrm{Case}-I\\ {\tilde{g}}_{2}z_{2}u, & \mathrm{Case}-\mathrm{II}\\ \sigma \left|z_{2}\right|{\tilde{g}}_{2}(g_{2}-g_{2N}), & \mathrm{Case}-\mathrm{III} \end{array}}}\;$$

Step 1: Considering the second term on the right-hand side of Equation (21), we have:

(22) $$\begin{array}{c} -(c_{2}z_{2}^{2}-z_{2}\varepsilon_{d})=-\frac{1}{2}c_{2}z_{2}^{2}-\frac{c_{2}}{2}{\left(z_{2}-\frac{\varepsilon_{d}}{c_{2}}\right)}^{2}+\frac{\varepsilon_{d}^{2}}{2c_{2}}\\ \leq -\frac{1}{2}c_{2}z_{2}^{2}+\frac{\varepsilon_{d}^{2}}{2c_{2}}\\ \leq -\frac{1}{2}c_{2}z_{2}^{2}+\frac{\varepsilon_{M}^{2}}{2c_{2}} \end{array}$$

Step 2: As to the third term, the following inequation can be obtained:

(23) $$\begin{array}{cc} \nu \left|z_{2}\right|{\tilde{\omega }}^{T}\hat{\omega } & =\nu \left|z_{2}\right|{\tilde{\omega }}^{T}\left(\omega^{*}-\tilde{\omega }\right)\\ & =-\nu \left|z_{2}\right|{\tilde{\omega }}^{T}\tilde{\omega }+\nu \left|z_{2}\right|{\tilde{\omega }}^{T}\omega^{*}\\ & \leq -\nu \left|z_{2}\right|{\left\|\tilde{\omega }\right\|}_{2}^{2}+\nu \left|z_{2}\right|{\left\|\tilde{\omega }\right\|}_{2}{\left\|\omega^{*}\right\|}_{2}\\ & \leq -\frac{1}{2}\nu \left|z_{2}\right|{\left\|\tilde{\omega }\right\|}_{2}^{2}+\frac{1}{2}\nu \left|z_{2}\right|{\left\|\omega^{*}\right\|}_{2}^{2}\\ & \leq -\frac{1}{2}\nu \left|z_{2}\right|{\left\|\tilde{\omega }\right\|}_{2}^{2}+\frac{1}{2}\nu \left|z_{2}\right|\omega_{M} \end{array}$$

Step 3: The fourth term is a piecewise function of three segments (Case-I, -II, -III). For Case-III, we have:

(24) $$\begin{array}{l} \sigma \left|z_{2}\right|{\tilde{g}}_{2}(g_{2}-g_{2N})\\ =\sigma \left|z_{2}\right|{\tilde{g}}_{2}\left(-{\tilde{g}}_{2}+g_{2}-g_{2N}\right)\\ =\sigma \left|z_{2}\right|\left\{-\frac{1}{2}{\tilde{g}}_{2}^{2}-\frac{1}{2}\left[{\tilde{g}}_{2}^{2}-2{\tilde{g}}_{2}\left(g_{2}-g_{2N}\right)+{\left(g_{2}-g_{2N}\right)}^{2}\right]+\frac{1}{2}{\left(g_{2}-g_{2N}\right)}^{2}\right\}\\ \leq -\frac{1}{2}\sigma \left|z_{2}\right|{\tilde{g}}_{2}^{2}+\frac{1}{2}\sigma \left|z_{2}\right|{\left(g_{2}-g_{2N}\right)}^{2} \end{array}$$

For Case-I: if ${\hat{g}}_{2}\geq {\bar{g}}_{2}$ and $\eta z_{2}u-\eta \sigma \left|z_{2}\right|\left({\hat{g}}_{2}-g_{2N}\right)\geq 0$, we know the following inequations:

(25) $$\begin{array}{l} {\hat{g}}_{2}\geq {\bar{g}}_{2}\geq g_{2N}\\ {\hat{g}}_{2}\geq {\bar{g}}_{2}\geq g_{2}\\ \;z_{2}u\geq 0\\ \;{\tilde{g}}_{2}\leq 0\\ \;{\tilde{g}}_{2}z_{2}u\leq 0 \end{array}$$

For Case-II, it is similar to that of Case-I. It is easy to prove that Inequation (25) still holds.

Note that if ${\hat{g}}_{2}\left(t_{0}\right)\in \left[{\underline{g}}_{2},{\bar{g}}_{2}\right]$, ${\underline{g}}_{2}\leq {\hat{g}}_{2}\leq {\bar{g}}_{2}$ is guaranteed by the projection operator [20].

Step 4: Case-III

Combining (21), (22), (23), and (24), we have:

(26) $$\begin{array}{cc} \dot{V} & \leq -c_{1}z_{1}^{2}-\frac{1}{2}c_{2}z_{2}^{2}+\frac{\varepsilon_{M}^{2}}{2c_{2}}-\frac{1}{2}\nu \left|z_{2}\right|{\left\|\tilde{\omega }\right\|}_{2}^{2}+\frac{1}{2}\nu \left|z_{2}\right|\omega_{M}\\ & -\frac{1}{2}\sigma \left|z_{2}\right|{\tilde{g}}_{2}^{2}+\frac{1}{2}\sigma \left|z_{2}\right|{\left(g_{2}-g_{2N}\right)}^{2} \end{array}$$

If $\left|z_{2}\right|=0$, Equation (26) can be reduced to:

(27) $$\dot{V}\leq -c_{1}z_{1}^{2}+\frac{\varepsilon_{M}^{2}}{2c_{2}}.$$

According to LaSalle’s invariance principle, $z_{1}$ is bounded. If ${\hat{g}}_{2}\left(t_{0}\right)$ and $\hat{\omega }\left(t_{0}\right)$ are bounded, ${\hat{g}}_{2}$ and $\hat{\omega }$ are bounded.

If $\left|z_{2}\right|\neq 0$, Equation (26) can be rewritten as the following form:

(28) $$\dot{V}\leq -cV+\rho ,$$

where

$$c=\min\left(c_{1},\frac{c_{2}}{2},\frac{\nu }{2}\left|z_{2}\right|,\frac{\sigma }{2}\left|z_{2}\right|\right),$$

$$\rho =\frac{\varepsilon_{M}^{2}}{2c_{2}}+\frac{\nu }{2}\left|z_{2}\right|\omega_{M}+\frac{\sigma }{2}\left|z_{2}\right|{\left(g_{2}-g_{2N}\right)}^{2}.$$

Therefore, V converges exponentially until $V\left(z_{1},z_{2},{\hat{g}}_{2},\hat{\omega }\right)\leq \rho /c$ [32].

Case-I and Case-II:

Combining (21), (22), (23), and (25), we have:

(29) $$\dot{V}\leq -c_{1}z_{1}^{2}-\frac{1}{2}c_{2}z_{2}^{2}+\frac{\varepsilon_{M}^{2}}{2c_{2}}-\frac{1}{2}\nu \left|z_{2}\right|{\left\|\tilde{\omega }\right\|}_{2}^{2}+\frac{1}{2}\nu \left|z_{2}\right|\omega_{M}.$$

Similar to Case-III, the boundedness of all closed-loop signals and errors could still hold.

Conclusion: Considering the system (4), control law (14), and adaptive laws (15) and (18) under Assumption 1, all signals of the closed-loop system ((4), (14), (15), and (18)) are bounded.

**Remark** **2.** *The steady state tracking error*$\left[z_{1},z_{2}\right]$*can be arbitrarily small by increasing the control gain*$\left(c_{1},c_{2}\right)$*and elaborately choosing the design value of*l*,*γ*,*ν*,*η*, and*σ*[33]*.

## 4. Simulation and Discussion

**Figure 7:**
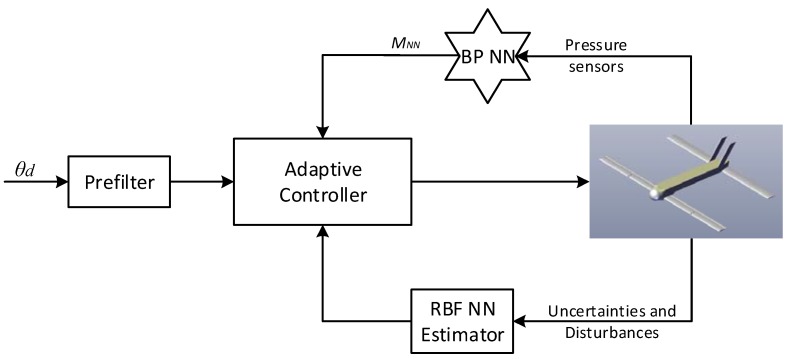
Block diagram of the bio-inspired control system. RBF: radial basis function.

In this section, the derived bio-inspired neural adaptive control (BNAC) structure was tested by simulation in MATLAB^®^. Figure 7 shows the block diagram of the system. In order to verify its performance and effectiveness, the closed-loop system—while suffering external disturbance, measurement noise, and parametric uncertainties—was simulated to track a pre-filtered 5° step command. Four representative simulation cases were presented. The initial states were set as: $V_{0}$ = 25 m/s, $\theta_{0}$ = 0°. The number of RBF NN hidden layer nodes was chosen as five. The design parameters were designed as: γ=12, η=6, ν=0.05, σ=0.05, $c_{1}=20$, and $c_{2}=20$, respectively. The actuator was modeled as a second-order system with a damping ratio of 0.8 and a natural frequency of 72. The external disturbance torque with an amplitude of 0.5 Nm was assumed to occur at 10 s. The gyro outputs were assumed to be polluted by white noise with mean of 0 and 0, and standard deviation of 0.21 °/s and 0.62 °/s^2^, respectively. The pressure sensors were selected with 95% accuracy of±15 Pa. Pressure measurements were filtered by low-pass filters with a time constant of 0.08 s.

For demonstrative purposes, the BNAC was compared with the basic backstepping controller and a widely used cascaded PID controller. The control gains $c_{1}$ and $c_{2}$ of the compared basic backstepping controller were the same as BNAC. The PID controller had a classical structure of two loops—a pitching rate inner loop and a pitch angle outer loop. On the basis of the frequency response of the SUAV and the actuator, the inner-loop system was tuned to a cut-off frequency of 15 and a phase margin of 60°. The outer loop was tuned to a cut-off frequency of 10 and a phase margin of 83°. The PID parameters are shown in Table 4.

**Figure 8:**
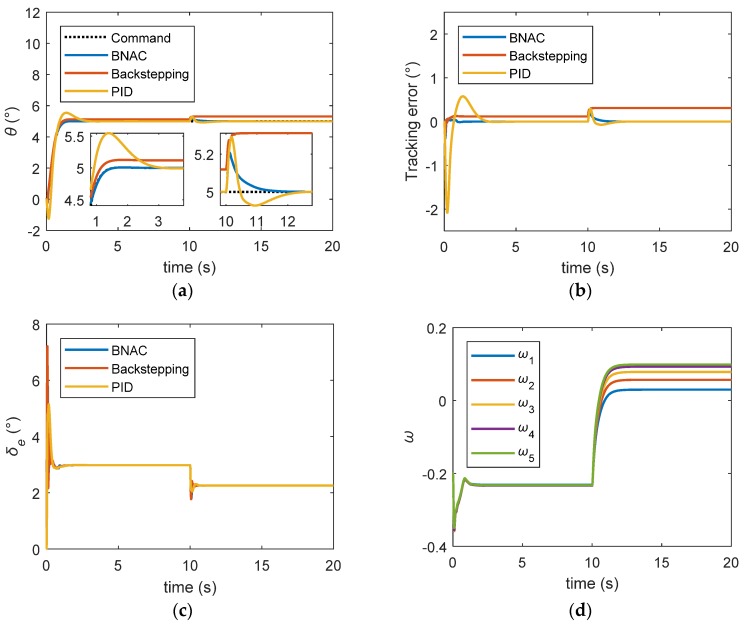
Step signal tracking results under external disturbance: (**a**) pitch angle trajectory; (**b**) tracking error; (**c**) elevator deflection; (**d**) RBF NN weights. BNAC: bio-inspired neural adaptive control. (BNAC: bio-inspired neural adaptive control, PID: proportion integration differentiation)

Figure 8 shows the control performance of the nominal model when no measurement noise was assumed. From time = 0 s to time = 10 s, the transient responses of the basic backstepping controller and BNAC were satisfactory. The PID controller overshot by about 10%. The pitch angles soon became stable. The tracking error of BNAC quickly converged to zero, while the PID required 2 s. There was a steady state error of the backstepping controller. To test the disturbance rejection performance of the controllers, the external disturbance torque was injected into the model between 10 and 20 s. BNAC could still maintain a good tracking performance when the external disturbance occurred. Because of the integrator, the PID controller withstood the disturbance and the output finally approached the command signal. It can be concluded that the BNAC performed better than the backstepping and PID controller under external disturbances, as shown in Figure 8b.

**Figure 9:**
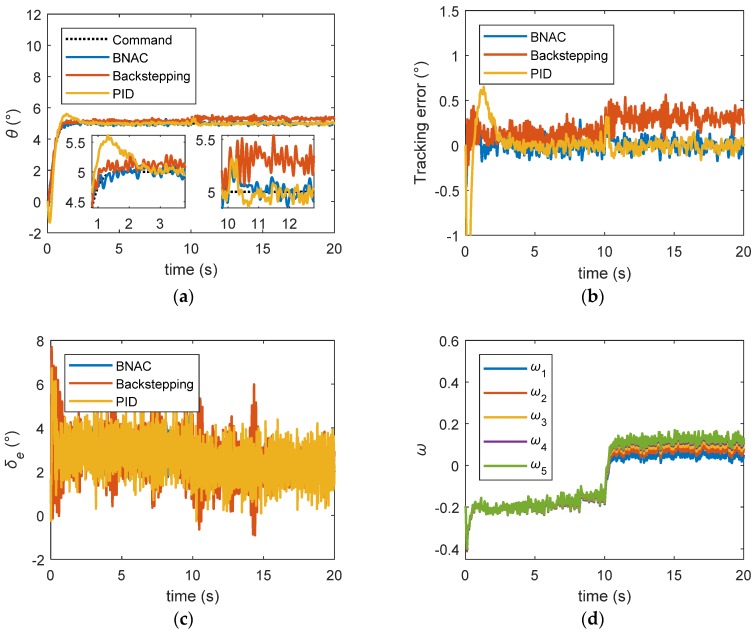
Step signal tracking results under external disturbance and noise: (**a**) pitch angle trajectory; (**b**) tracking error; (**c**) elevator deflection; (**d**) RBF NN weights.

The tracking performances with respect to measurement noise are shown in Figure 9. The backstepping controller had the worst robustness and anti-noise ability. The PID controller had a good anti-noise performance but the worst transient performance, although the PID controller had the advantage of being cheaper and easier to implement.

**Figure 10:**
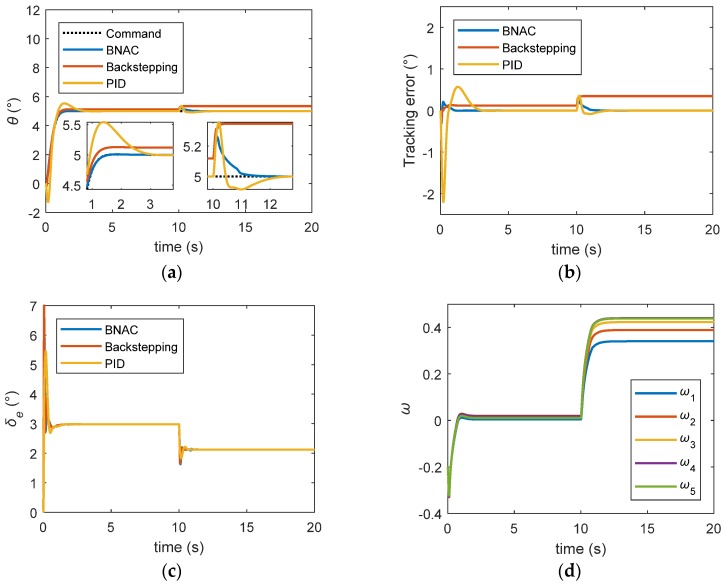
Step signal tracking results under external disturbance and parametric uncertainties: (**a**) pitch angle trajectory; (**b**) tracking error; (**c**) elevator deflection; (**d**) RBF NN weights.

**Figure 11:**
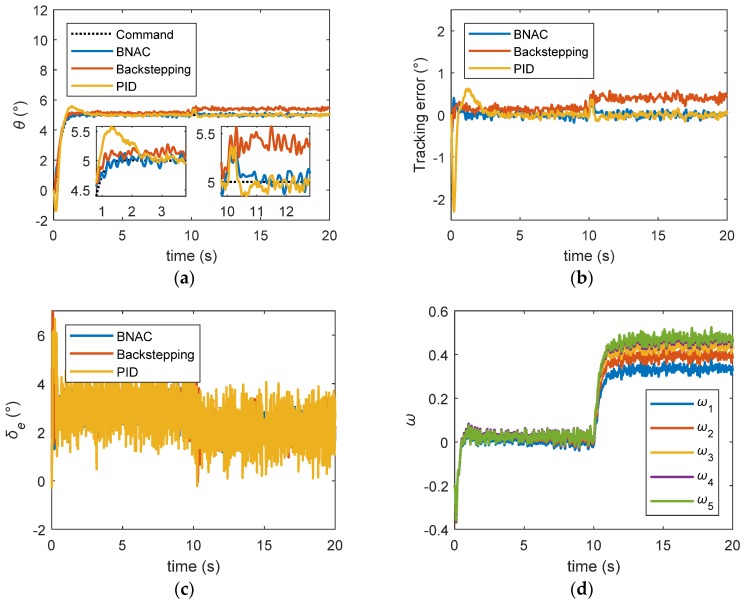
Step signal tracking results under external disturbance, measurement noise, and parametric uncertainties: (**a**) pitch angle trajectory; (**b**) tracking error; (**c**) elevator deflection; (**d**) RBF NN weights.

Figure 10 and Figure 11 demonstrate the robustness with respect to parametric uncertainties. For the sake of performance comparison, only three kinds of uncertain parameters were considered: reference area $S_{ref}$, moment of inertia $I_{yy}$, and air density $\rho_{air}$. The maximum values of the additive uncertainties were taken as follows:

|ΔSrefSref|≤0.06, |ΔIyyIyy|≤0.05, |Δρairρair|≤0.1.

Figure 10 clearly shows that the BNAC structure had a good performance and exhibited robustness when subjected to parametric uncertainties and external disturbance. Figure 11 demonstrates the exceptional quality of the proposed BNAC system to accurately track command when measurement noise, parametric uncertainties, and external disturbances occurred.

## 5. Conclusions

This paper proposes a bio-inspired neural adaptive SUAV control system. Pressure sensors were embedded in the forward wing surface to sense the airflow around the vehicle. The sensor locations elaborately designed. A BP NN was used to estimate the aerodynamic moment based on the pressure information. The SUAV’s aerodynamic model was modified to match the control system. To make full use of the estimation, a robust neural adaptive controller was proposed, based on a RBF NN. The closed-loop stability was analyzed via Lyapunov theory. The derived robust adaptive controller was tested by simulation. The simulation results showed a good performance of the proposed SUAV control system to accurately track command when measurement noise, parametric uncertainties, and external disturbance occurred. Future works will focus on: (1) improving the estimator performance, (2) expanding the controller to 3-axis attitude, and (3) implementing flight experiments.

## Figures and Tables

**Table 1:** Pressure sensor location in the front wing.

Pressure Sensor	Chordwise (x/Chord)	Spanwise (y/Span)
P1, P5	0.05	1/4, 3/4
P2, P6	0.35	1/4, 3/4
P3, P7	0.1	1/4, 3/4
P4, P8	0.35	1/4, 3/4

**Table 2:** Computational aerodynamic data samples: sampling rate of 2°.

*α* (°)	P1 (Pa)	P2 (Pa)	P3 (Pa)	P4 (Pa)	P5–P8	M (Nm)
−6	101,539	101,333	100,734	101,172	…	−2.80
−4	101,471	101,297	101,013	101,195	…	−2.78
−2	101,382	101,259	101,105	101,233	…	−2.74
0	101,275	101,218	101,191	101,269	…	−2.55
2	101,154	101,177	101,273	101,305	…	−2.26
4	101,018	101,136	101,348	101,339	…	−2.11
6	100,870	101,094	101,417	101,372	…	−2.02
8	100,714	101,052	101,478	101,403	…	−1.93
10	100,557	101,010	101,531	101,433	…	−1.81
12	100,414	100,969	101,578	101,461	…	−1.54

**Table 3:** The index of 36 test samples.

***α* (1–10°)**	−5.58	−5.10	−4.98	−4.39	−3.56	−3.16	−2.69	−2.10	−1.61	−1.43
***α* (11–20°)**	−0.55	−0.01	0.12	0.93	1.16	1.64	2.27	2.69	3.07	3.74
***α* (21–30°)**	4.40	4.78	5.27	5.62	6.46	6.88	7.08	7.73	8.31	8.54
***α* (31–36°)**	9.11	9.63	10.13	10.71	11.13	11.94	×	×	×	×

**Table 4:** The PID controller parameters.

Inner Loop PID			Outer Loop PID		
*K_p_*	*K_i_*	*K_d_*	*K_p_*	*K_i_*	*K_d_*
0.12	1.1	0.0034	6.0	9.4	0.47

## Abbreviations

The following abbreviations are used in this manuscript:

SUAV	small unmanned aerial vehicle
UAV	unmanned aerial vehicle
IMU	inertial measurement unit
BP	back propagation
RBF	radial basis function
NN	neural network
CFD	computational fluid dynamics
BNAC	bio-inspired neural adaptive control
PID	proportion integration differentiation
